# When Isolated at Full Receptivity, *in Vitro* Fertilized Wheat (*Triticum aestivum*, L.) Egg Cells Reveal [Ca^2+^]_cyt_ Oscillation of Intracellular Origin

**DOI:** 10.3390/ijms151223766

**Published:** 2014-12-19

**Authors:** Zsolt Pónya, Ilaria Corsi, Richárd Hoffmann, Melinda Kovács, Anikó Dobosy, Attila Zoltán Kovács, Mauro Cresti, Beáta Barnabás

**Affiliations:** 1 Department of Plant Production and Plant Protection, Institute of Plant Science, Faculty of Agricultural and Environmental Sciences, Kaposvár University, Kaposvár H-7400, Hungary; E-Mails: hoffmann.richard@ke.hu (R.H.); aniko.dobosy@esolution.hu (A.D.); 2 Dipartimento di Scienze Ambientali “G. Sarfatti”, University of Siena, Siena 53100, Italy; E-Mails: ilaria.corsi@unisi.it (I.C.); cresti@unis.it (M.C.); 3 Institute of Physiology, Biochemistry and Animal Health, Faculty of Agricultural and Environmental Sciences, Kaposvár University, Kaposvár H-7400, Hungary; E-Mail: kovacs.melinda@ke.hu; 4 Department of Technology of Animal Breeding and Management, Faculty of Agricultural and Environmental Sciences Kaposvár University, Kaposvár H-7400, Hungary; E-Mail: kovacs.attila@ke.hu; 5 Department of Plant Cell Biology, Agricultural Institute, Centre for Agricultural Research, Hungarian Academy of Sciences, Martonvàsàr H-2462, Hungary; E-Mail: barnabas.beata@agrar.mta.hu

**Keywords:** *in vitro* fertilization, wheat (*Triticum aestivum*, L.) egg cell, cytosolic calcium, egg activation, thapsigargin, intracellular Ca^2+^ store, endoplasmic reticulum

## Abstract

During *in vitro* fertilization of wheat (*Triticum aestivum*, L.) in egg cells isolated at various developmental stages, changes in cytosolic free calcium ([Ca^2+^]_cyt_) were observed. The dynamics of [Ca^2+^]_cyt_ elevation varied, reflecting the difference in the developmental stage of the eggs used. [Ca^2+^]_cyt_ oscillation was exclusively observed in fertile, mature egg cells fused with the sperm cell. To determine how [Ca^2+^]_cyt_ oscillation in mature egg cells is generated, egg cells were incubated in thapsigargin, which proved to be a specific inhibitor of the endoplasmic reticulum (ER) Ca^2+^-ATPase in wheat egg cells. In unfertilized egg cells, the addition of thapsigargin caused an abrupt transient increase in [Ca^2+^]_cyt_ in the absence of extracellular Ca^2+^, suggesting that an influx pathway for Ca^2+^ is activated by thapsigargin. The [Ca^2+^]_cyt_ oscillation seemed to require the filling of an intracellular calcium store for the onset of which, calcium influx through the plasma membrane appeared essential. This was demonstrated by omitting extracellular calcium from (or adding GdCl_3_ to) the fusion medium, which prevented [Ca^2+^]_cyt_ oscillation in mature egg cells fused with the sperm. Combined, these data permit the hypothesis that the first sperm-induced transient increase in [Ca^2+^]_cyt_ depletes an intracellular Ca^2+^ store, triggering an increase in plasma membrane Ca^2+^ permeability, and this enhanced Ca^2+^ influx results in [Ca^2+^]_cyt_ oscillation.

## 1. Introduction

In the eggs of all animal species studied so far, fertilization induces an increase in cytosolic calcium ([Ca^2+^]_cyt_), which appears to be the primary intracellular signal responsible for the initiation of the development of the egg following fertilization [1,2,3,4,5,6]. Thus, the fertilizing spermatozoon triggers a common cascade of events by generating [Ca^2+^]_cyt_ transients in the cytoplasm of the egg [7,8,9,10]. Although the pattern of this [Ca^2+^]_cyt_ varies, the pulsatory rise in [Ca^2+^]_cyt_ seems to be a universal phenomenon that marks the onset of egg activation among the mammalian species investigated thus far [11,12,13,14,15,16]. In sea urchin, however, a single, transient [Ca^2+^]_cyt_ rise induced by fertilization was reported [17,18,19]. Therefore, one of the earliest events that occurs in the animal egg during fertilization is at least one increase in [Ca^2+^]_cyt_ [20,21,22]. Furthermore, development of an inositol trisphosphate (InsP_3_)-induced calcium release mechanism during maturation of hamster oocytes has been demonstrated by Miyazaki* et al.* [13] and Fujiwara* et al.* [15]. From these studies, it seems to be clear that the release of Ca^2+^ from intracellular stores is mediated by InsP_3_ through the opening of InsP_3_-activated Ca^2+^-channels (InsP_3_receptors) on the endoplasmic reticulum. 

Much less is known, however, about calcium signaling during egg activation in higher plants. This is mainly due to the inaccessibility of the female gametophyte for experimental manipulation, which makes the cellular/molecular study of fertilization-associated events in higher plants difficult (for a review, see [23,24]). Nonetheless, recently developed techniques, such as gamete isolation and* in vitro* fertilization of gamete pairs, offer the possibility of studying the first events associated with gamete fusion (for a review, see [25]). Exploiting a calcium-induced* in vitro* fertilization system, Digonnet* et al.* [26] reported first a fertilization-associated Ca^2+^ transient in the cytoplasm of the fertilized maize egg. Furthermore, recently, the protein, annexin p35, was identified in the egg cell and zygote of maize and shown to be involved in the exocytosis of cell wall materials (an important event during the development of the fertilized egg cell), which was found to be induced by a fertilization-triggered increase in cytosolic Ca^2+^ levels [27]. These findings suggested that egg activation in higher plants may involve mechanisms similar to those that had been found to act in mammalian fertilization and in that in a brown alga, *Fucus* (Phaeophyceae) [28,29].

Capitalizing on the Ca^2+^-selective vibrating electrode method, Antoine* et al.* [30] observed a Ca^2+^ influx spreading through the entire plasma membrane of the maize egg cell fertilized* in vitro* by using extracellular calcium. In this study, however, the introduction of the so-called calcium-sensitive ratio dyes into the egg’s cytoplasm, which would allow for precisely following the spatial and temporal changes in [Ca^2+^]_cyt_, was not possible, due to the failure of injecting the delicate egg cells, hence leaving important questions, such as the origin and the dynamics of the observed calcium signal, unanswered [31].

In the present study, dual-ratio imaging of cytosolic calcium [Ca^2+^]_cyt_ was performed in order to investigate the characteristics of the calcium signal during fertilization in the wheat female gamete. Employing a microinjection technique elaborated by Pónya* et al.* [32] allowed for the injection of isolated wheat (*Triticum aestivum*, L.) egg cells with the calcium-sensitive ratio dye (fura-2 dextran) in liquid medium, thus making IVF (*in vitro* fertilization) possible following injection. This method was combined with the electrofusion procedure elaborated by Kranz* et al.* [33] for maize gamete fusion [33,34]. Combining these two techniques made it possible to gain quantitative data on the duration, amplitude and frequency of the [Ca^2+^]_cyt_ changes observed in the fertilized wheat egg, which permits quantitative comparisons to be made between the characteristics of the calcium signal ensuing upon fertilization in the animal egg and in the female gamete of wheat, a higher land plant. In view of the structural changes that the ER goes through during the* in situ* development of the wheat egg [35], which could be correlated with a change in the calcium storage capacity of the ER and based on the observation made by Pónya* et al.* [36] that in the receptive wheat egg cell the main calcium store is the endoplasmic reticulum (ER), the dynamics of changes in [Ca^2+^]_cyt_ in wheat female gametes isolated at different maturational stages and fertilized* in vitro* were followed. Egg protoplasts were isolated at different developmental stages defined according to the time (measured as days after emasculation; DAE) elapsed from emasculation, carried out at a certain developmental window of the male gametophyte. Three maturational windows were defined for the female gametes to be isolated for the experiments: (1) three DAE, at which isolated eggs were considered immature; (2) six DAE, yielding mature, receptive eggs; and (3) 11 DAE, the isolation of overmature female gametes.

The advantage of electrofusion,* i.e.*, unlike the calcium-induced gamete fusion system [34], fusion is possible in calcium-free medium, was exploited to determine if and how intracellular calcium stored in intracellular calcium stores in the wheat egg plays a role in calcium signaling during fertilization with respect to the presence or omission of extracellular calcium in the fusion medium. For this purpose, IVF was carried out either in Ca^2+^-free fusion medium or in fusion medium containing CaCl_2_.

Based on previous findings of Pónya* et al.* [35] that the mature wheat egg has only a few vacuoles and an extensive, well-developed endoplasmic reticulum (ER) system shown by Pónya* et al.* [36] to be the main intracellular Ca^2+^ store in the female gamete of wheat and also on the preliminary result that [Ca^2+^]_cyt_ elevation was also seen in egg cells incubated and fused in Ca^2+^ free medium (therefore, the calcium rise that was observed needed to have originated from an internal calcium store), the ER was assumed to be the origin of the repetitive [Ca^2+^]_cyt_ transients observed in mature, fertilized wheat (*T. aestivum*, L.) egg cells. To test this hypothesis, a pharmacological approach was employed to examine the origin of the fertilization-associated [Ca^2+^]_cyt_ change in the egg cytoplasm. Wheat female gametes were treated with thapsigargin, an inhibitor of Ca^2+^-pumps [37,38], which proved to be able to specifically block Ca^2+^-ATPases in the ER, while leaving the plasma membrane calcium-pumps unaffected, at least at a certain concentration (10 μM) of the drug added to the fusion medium.

## 2. Results

### 2.1. Imaging [Ca^2+^]_cyt_ during in Vitro Fertilization (IVF) of Isolated Egg Cells Developed in Situ

The possibility of the injection of fura-2 dextran into egg cells isolated from wheat allowed for the dual-excitation-based ratio approach to be used to measure [Ca^2+^]_cyt_ changes in the cytoplasm of the* in vitro* fertilized female gamete.

**Figure 1:**
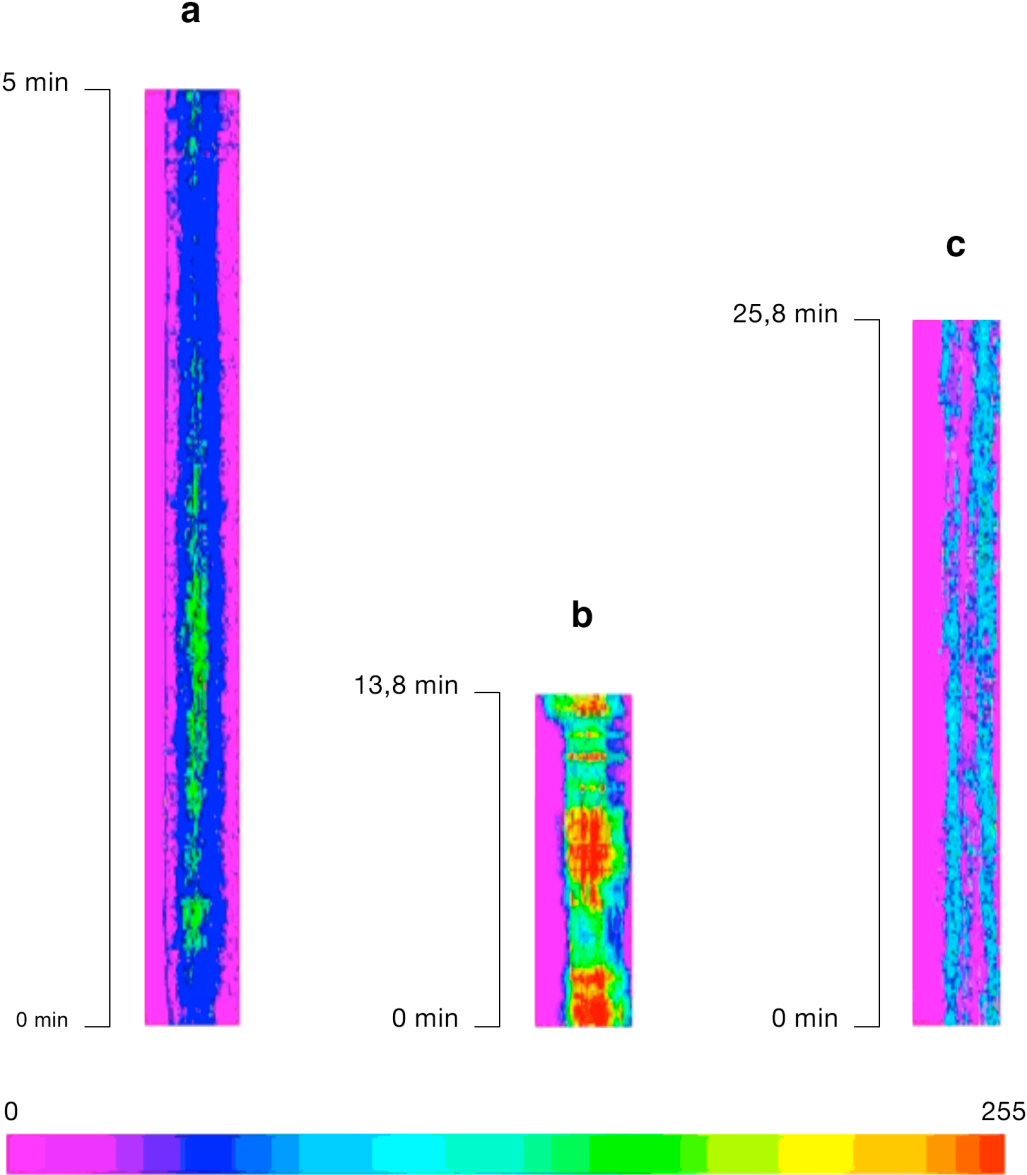
Calcium dynamics in reconstructions of temporal sections obtained with the Line Image function of the Lucida software. The line trace plot is represented as pixel intensities converted into pseudocolor values in the Line Image. Rows in the Line Image correspond to successive images (*x*) along the active dimension, time (*t*). Thus, the Line Image is an (*x*,*t*) plot showing [Ca^2+^]_cyt_ change along an axis through the “stack” image composed of the overlaid images taken successively during [Ca^2+^]_cyt_ measurement. The increase in [Ca^2+^]_cyt_ is represented by yellow-red bands. The bar represents a pseudocolor code of the pixel values digitized to 256 grey levels. (**a**) Line Image of an egg cell isolated three DAE, injected with fura-2 dextran and ratio-imaged following electrofusion with a sperm cell. The axis along which the [Ca^2+^]_cyt_ changes were measured passed through the sperm entry site; (**b**) [Ca^2+^]_cyt_ changes over time in a receptive egg cell (isolated six DAE) microinjected with fura-2 dextran and fertilized* in vitro*; (**c**) Time-lapse series of an axis “drawn” through time, the active dimension, in an overmature (18 DAE) egg cell fertilized* in vitro* following fura-2 dextran injection.

First, the [Ca^2+^]_cyt_ response of immature egg cells isolated three days after emasculation (DAE) (*i.e.*, shortly after the third mitosis of the female gametophyte was completed) to sperm incorporation was investigated. During the recording period, [Ca^2+^]_cyt_ did not rise above the basal level estimated by applying the GPT transformation (Grynkiewicz, Poene and Tseng calibration for calcium ion concentration with fluorescence ratio dyes) [39] to the fluorescence ratio image sequences (*n* = 36). As shown in Figure 1a, [Ca^2+^]_cyt_ rose only slightly above the basal level measured along an axis passing through the sperm entry site in immature egg cells isolated three DAE, whereas in Figure 1b, distinct (red) bands indicate the pulsatile elevations of [Ca^2+^]_cyt_ in a receptive egg cell (irrespective of whether the axis along which the measurement was taken passed through the sperm entry site or through the region of origin of [Ca^2+^]_cyt_ rise); whereas no [Ca^2+^]_cyt_ elevation could be detected in overmature egg cells isolated 18 DAE (*n* = 17) (Figure 1c).

**Figure 2:**
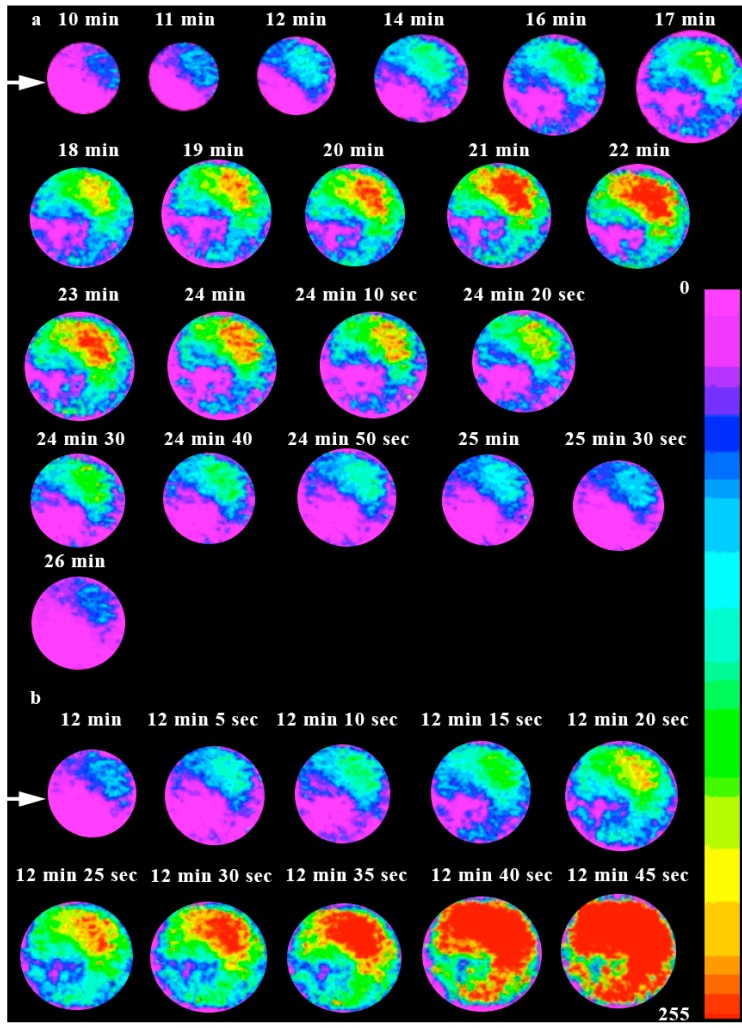
[Ca^2+^]_cyt_ ratio-imaging in immature and mature wheat egg cells at the focal plane of sperm incorporation. (**a**) The rise of a truncated [Ca^2+^]_cyt_ transient confined to a distinct region of the cytoplasm of the female gamete of wheat isolated three DAE and fused with a sperm cell. [Ca^2+^]_cyt_ elevation ensued approximately 10 min after sperm–egg fusion. Note that the site of the origin of the [Ca^2+^]_cyt_ transient is away from the fusion site (indicated by the arrows) of the male gamete and that the diameter of the pseudocolor-coded image sequences changes, so as to enhance the representation of the change in [Ca^2+^]_cyt_ elevation in such a way that the larger the diameter of the image, the higher the [Ca^2+^]_cyt_ concentration; the arrow indicates the sperm entry site; (**b**) [Ca^2+^]_cyt_ wave of a mature (six DAE) wheat egg cell sweeping through the whole cytoplasm of the cell approximately 12 min after plasmogamy. Note that the origin of the [Ca^2+^]_cyt_ wave is away from the sperm entry site. The arrow shows the sperm entry site.

In the time series of ratio [Ca^2+^]_cyt_ images shown in Figure 2a,b, changes in [Ca^2+^]_cyt_ were observed to arise away from the sperm entry site. In the case of immature egg cells, this rise in [Ca^2+^]_cyt_ was confined to a certain region of the cell away from the sperm entry site, whereas fully mature egg cells exhibited [Ca^2+^]_cyt_ waves sweeping through the entire cell at the focal plane of sperm entry (Figure 2a, Figure 2b).

**Figure 3:**
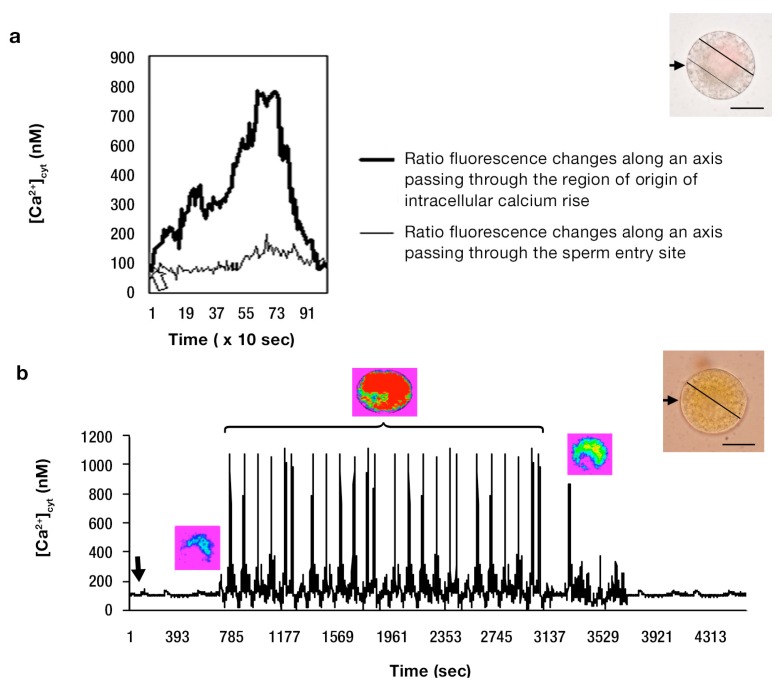

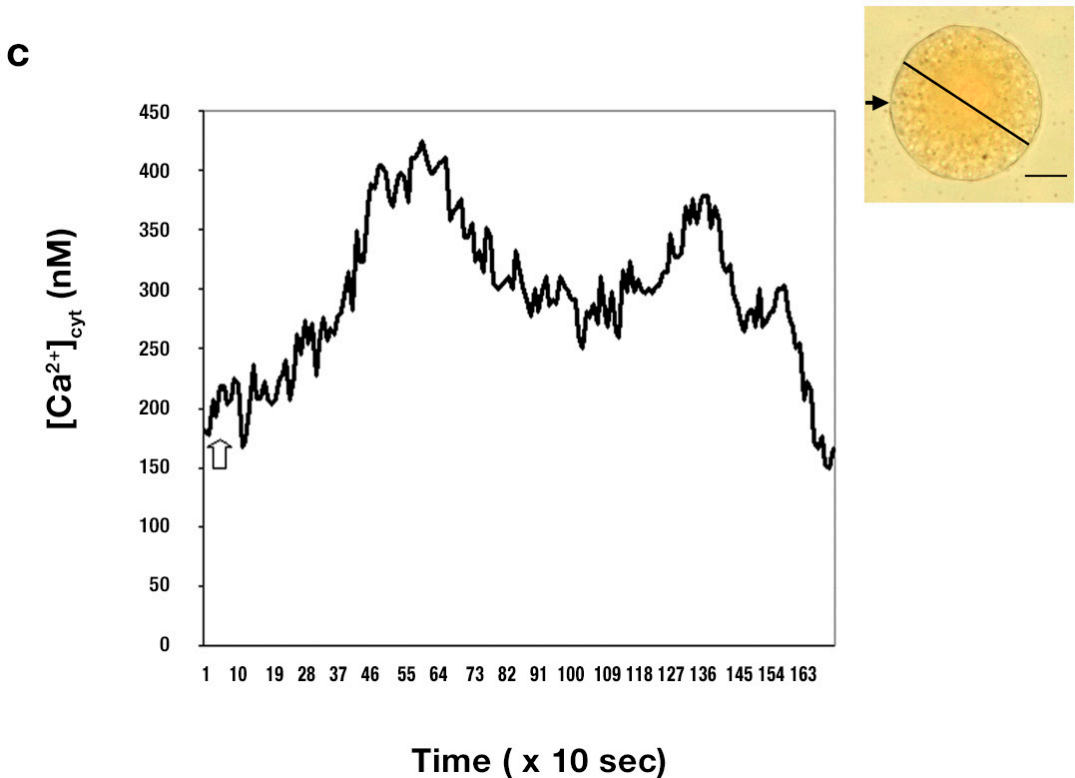
The 340/380 nm excitation ratios of fura-2 dextran-injected wheat egg cells showing [Ca^2+^]_cyt_ variations in response to the different maturational stages of the batch of the egg cells used for* in vitro* fertilization. (**a**) Typical, fertilization-associated [Ca^2+^]_cyt_ rise in an* in vitro* fertilized wheat egg cell developed* in situ* and isolated three DAE (the arrow denotes 10 min after *in vitro* fertilization (IVF)). The bright field image (inset) at the right upper corner shows the lines (axes) of pixels along which the pixel intensities (*i.e*., the changes in calcium concentrations) were measured through the active dimension, time; the arrow shows the site of sperm incorporation, and the bar represents: 15 µm; (**b**) Representative [Ca^2+^]_cyt_ changes occurring concomitantly upon* in vitro* fertilization of mature wheat egg cells (isolated at six DAE). This dynamics of the [Ca^2+^]_cyt_ change could be seen in 66 out of 80 (81.5%) egg cells fertilized with the sperm (the arrow indicates the time at which fusion between the sperm and the egg cell occurred). The [Ca^2+^]_cyt_ peak elicited by the sperm ensued 10 min after the* in vitro* fusion of the gametes of opposite sexes. The pseudo-colored images (insets) give a visual representation of the change in [Ca^2+^]_cyt_, whereas the bright-field image shows the axis along which the pixel intensities (*i.e*., the changes in calcium concentrations) were measured. The arrow shows the site of sperm entry, and the bar represents: 20 µm; (**c**) A slow [Ca^2+^]_cyt_ rise induced by sperm cell fusion in an overmature egg cell isolated 11 DAE (the arrow shows the time lapse, 17 min, between sperm–egg fusion and the commencement of the slow [Ca^2+^]_cyt_ elevation). The bright-field image at the right upper corner shows the axis along which the pixel intensities (*i.e*., the changes in calcium concentrations) were measured. The arrow shows the site of sperm entry, and the bar represents: 25 µm.

The finding that in the* in vitro* fertilized, immature egg protoplasts, the [Ca^2+^]_cyt_ rise was confined to a distinct region of the cytoplasm away from the site of sperm incorporation (see Figure 2a) was corroborated by calculating (using the Lucida software, Kinetic Imaging, Merseyside, UK) the average fluorescence intensity along an axis (drawn with the computer mouse) passing through the region of the origin of the rise in [Ca^2+^]_cyt_ across the time-series of the successive images. The quantitative data obtained were subsequently compared with those gained in the same way, but depicting average [Ca^2+^]_cyt_ changes along an axis passing through the sperm entry site (*i.e*., along an axis drawn through the time-series of the stack of the successive images, which did not pass through the origin of the [Ca^2+^]_cyt_ change (Figure 3a).

The resting level of [Ca^2+^]_cyt_ in unfertilized, mature wheat egg cells was estimated from the ratio equation and found to be 109 ± 27 nM (*n* = 66), which approximately 10 ± 2 min (*n* = 66) after gamete fusion rose to 1100 ± 21 nM (*n* = 66) as the highest [Ca^2+^]_cyt_ peak reached its summit, which was followed by a global elevation in [Ca^2+^]_cyt_, as was observed at the focal plane corresponding to the Sperm Entry Site (SES) throughout the whole cell (Figure 2b and Figure 3b). In receptive wheat (*T. aestivum*, L.) egg cells, the first [Ca^2+^]_cyt_ rise was typically followed by several [Ca^2+^]_cyt_ pulses, the oscillatory maximum of which was estimated to be 1180 ± 40 nM (*n* = 66), as the resting [Ca^2+^]_cyt_ level had increased about 13-fold when the [Ca^2+^]_cyt_ spikes reached their peak (Figure 3b). The magnitude of the average global [Ca^2+^]_cyt_ rise did not exceed 442 ± 15 nM (*n* = 66) and usually corresponded to about a 10-fold increase (1100 ± 21 nM) (*n* = 66) (Figure 3b). The calculated propagation velocity of the wave front was found to be 0.9 ± 0.4 µm/s (*n* = 66). A typical measurement of [Ca^2+^]_cyt_ in egg cells isolated at 11 DAE and fertilized with viable, mature sperm cells is depicted in Figure 3c. Isolated at this maturational stage, the fertilized egg protoplasts showed a delayed [Ca^2+^]_cyt_ rise compared to that of receptive eggs. The last developmental stage at which changes in [Ca^2+^]_cyt_ were elicited by the sperm cell in isolated egg cells was at 11 DAE. At this maturational stage, the [Ca^2+^]_cyt_ rise occurred 10 ± 3 min (*n* = 45) later than in mature, fertilized egg cells (isolated six DAE) and presented a slow rise of [Ca^2+^]_cyt_, which reached a plateau at 424 ± 17 nM (*n* = 45) with a mean amplitude of 295 ± 22 nM (*n* = 45) 27 min post-fertilization (Figure 3c). The egg cells remained at this [Ca^2+^]_cyt_ level for an additional 17 min, then at 44 ± 4 min (*n* = 45) after fusion, the level began to decrease, until [Ca^2+^]_cyt_ could not be distinguished from the basal level (Figure 3c). Sperm-induced [Ca^2+^]_cyt_ elevations characteristic of mature egg cells could not be observed in any of the cells (*n* = 45) isolated at this maturational stage.

**Figure 4:**
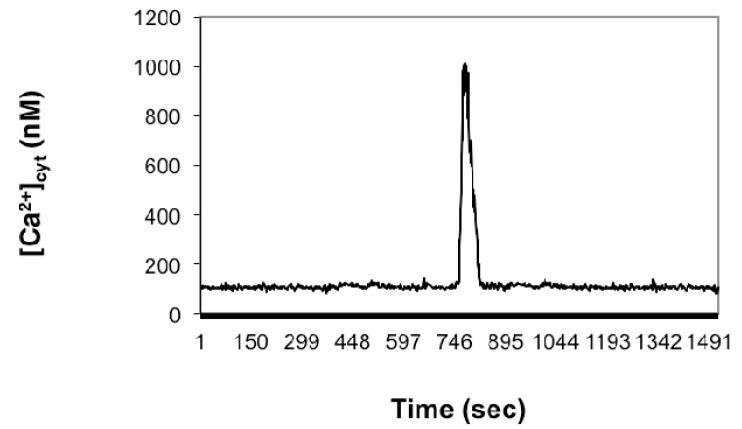
[Ca^2+^]_cyt_ dynamics in a receptive egg cell isolated six DAE and fertilized* in vitro* in fusion medium containing 10 µM (final concentration) of GdCl_3_. Time was measured from the successful incorporation of the sperm into the egg’s cytoplasm. The onset of the rise in [Ca^2+^]_cyt_ elicited by the sperm ensued 12 ± 1.4 min, *n* = 18, following* in vitro* fusion of the sperm cell with the female gamete.

In order to assess the contribution to the [Ca^2+^]_cyt_ dynamics of calcium influx across the plasma membrane of the mature egg cell, IVF was carried out in fusion medium without calcium or in IVF medium containing 10 µM (final concentration) of GdCl_3_, which had been previously demonstrated by Antoine* et al.* [30] to reproducibly and efficaciously inhibit Ca^2+^ influx in maize egg cells. As revealed by Figure 4, the secondary Ca^2+^ transients required extracellular Ca^2+^, because when sperm–egg cell fusion was performed in Ca^2+^-free IVF medium to which 10 µM (final concentration) of GdCl_3_ (widely used as an inhibitor of stretch-activated Ca^2+^-channels [30,40,41],) was added, no [Ca^2+^]_cyt_ oscillation could be detected (*n* = 18); instead, a single [Ca^2+^]_cyt_ rise occurred (Figure 4).

**Figure 5:**
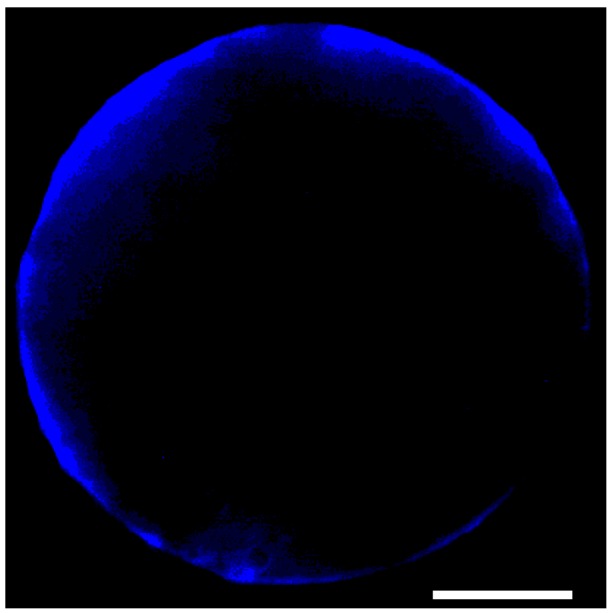
Cell wall regeneration in an egg protoplast fused* in vitro* with the sperm in Ca^2+^-free IVF medium. The image was taken 2 h after the sperm–egg cytoplasmic continuity had been established. Scale bar, 12.5 µm.

Suggesting that an increase in plasma membrane Ca^2+^ permeability is necessary for the onset of the [Ca^2+^]_cyt_ peaks (using 1,2-bis-/2-aminophenoxy/-ethane-*N*,*N*,*N*',*N*'-tetraacetic acid (BAPTA) to chelate any external Ca^2+^ was not feasible, since it caused the loss of sperm membrane integrity within minutes after the introduction of the compound into the IVF medium, a finding corroborating that of Antoine* et al.* [41]), this observation is in agreement with the results of Antoine* et al.* [30], who measured Ca^2+^ influx through the egg cell plasma membrane using the Ca^2+^-selective vibrating probe. Although these cells (13 out of 15) were capable of cell wall regeneration, as is revealed by Figure 5, no cell division could be observed during their* in vitro* culture.

A primary concern of the present study was to verify that the observed calcium rises had indeed physiological relevance in egg activation and in the continuation of the normal development of the fertilized egg; consequently, the measured changes in [Ca^2+^]_cyt_ do not reflect a putative stress response induced by the experimental procedures (such as egg cell isolation, incubation off the maternal tissue, “excess” extracellular Ca^2+^ present in the IVF medium or the microinjection/electrofusion procedures) in the egg. For this purpose, numerous control experiments were carried out, such as to demonstrate that impaling the fragile egg gametoplasts with the injection needle, injection itself and withdrawing the microcapillary did not elicit “artificial” changes in [Ca^2+^]_cyt_, nor did the electrofusion procedure (without the sperm cell) trigger events leading to [Ca^2+^]_cyt_ rise (see Figure S1a,b in the Supplementary). These control experiments unambiguously demonstrated that under our experimental conditions, it was possible to follow accurately the spatial-temporal changes in [Ca^2+^]_cyt_ measured in wheat egg cells fertilized* in vitro* and that the measured [Ca^2+^]_cyt_ changes are not due to stress responses, but indeed have physiological relevance to egg activation (see the Figures S1–S7 in the Supplementary).

### 2.2. Effect upon [Ca^2+^]_cyt_ of Thapsigargin Added to the IVF Medium

Based on previous findings [36], the endoplasmic reticulum (ER) was assumed to be the origin of the observed [Ca^2+^]_cyt_ rise in the fertilized egg cell. To test this hypothesis, thapsigargin, a tumor-promoting plant sesquiterpene lactone, was added to mature egg cells prior to and following* in vitro* fertilization, and its effect on [Ca^2+^]_cyt_ dynamics was studied. Thapsigargin was previously shown to inhibit animal intracellular SERCA-type Ca^2+^ pumps present in the sarcoplasmic/endoplasmic reticulum [38,42] and found to have an inhibitory effect on calcium pumps residing in the ER and the plasma membrane (PM) in red beet [43].

**Figure 6:**
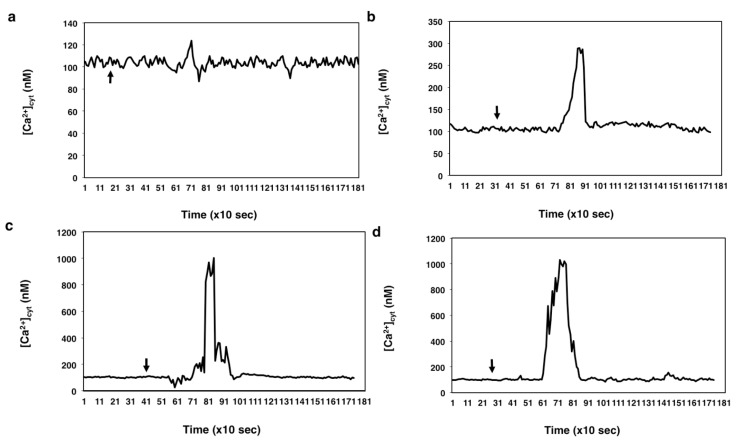
The effect of thapsigargin on [Ca^2+^]_cyt_ measured in unfertilized egg cells incubated in calcium-free IVF medium. Thapsigargin was added to unfertilized eggs at: 0.1 μM (**a**), 1 μM (**b**), 10 μM (**c**) and at 50 μM (**d**). The arrows designate the times of the addition of the drug.

The ability of thapsigargin to deplete the calcium pumps in the wheat egg appeared to be concentration dependent, since the drug produced varying degrees of [Ca^2+^]_cyt_ elevation when applied at different concentrations (Figure 6a–d).

When applied at a 10 μM concentration, thapsigargin appeared to induce calcium release from an intracellular calcium store(s) in the wheat egg (*n* = 27). In unfertilized egg cells that were incubated in Ca^2+^-free IVF medium, the drug triggered a single Ca^2+^ peak with as high an amplitude as that caused by the sperm cell (Figure 6c). Increasing the concentration of the drug from 10 to 50 μM did not cause higher elevation in [Ca^2+^]_cyt_ (1003 ± 11 nM, *n* = 27; 1003 ± 16, *n* = 22, respectively) compared to that triggered by incubating the cells in 10 μM thapsigargin, which suggests total depletion of the calcium pumps of the ER by thapsigargin added at 10 μM concentration to the cells (compare Figure 6c,d).

**Figure 7:**
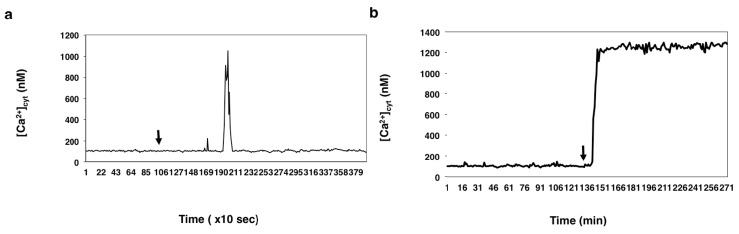
The effect of thapsigargin added at (**a**) 10 µM and at (**b**) 100 µM concentration on the cytosolic calcium level of unfertilized wheat egg cells incubated in IVF medium containing 2 mM CaCl_2_. Arrows: addition of thapsigargin.

When the female gametoplasts were incubated in IVF medium containing 2 mM CaCl_2_, the drug, present at the same concentration (10 μM), caused a single [Ca^2+^]_cyt_ rise, the peak value (1049 ± 13 nM, *n* = 15) of which was not significantly different from that observed when the cells were incubated in calcium-free IVF medium (1003 ± 11 nM, *n* = 27) (compare Figure 6c and Figure 7a), suggesting that at this concentration, thapsigargin does not exert an inhibitory effect on the plasma membrane calcium pumps.

However, the addition of 100 μM thapsigargin to unfertilized egg protoplasts incubated in IVF medium containing calcium (2 mM CaCl_2_) induced a [Ca^2+^]_cyt_ rise, the peak value (1300 ± 22 nM, *n* = 17) of which was higher than that (1049 ± 13 nM, *n* = 15) produced by thapsigargin applied at a 10 μM concentration (compare Figure 7a,b) to the egg cells under the same conditions. This increase in [Ca^2+^]_cyt_ was sustained and in none of the egg cells (*n* = 15) analyzed returned to the basal [Ca^2+^]_cyt_ level.

**Figure 8:**
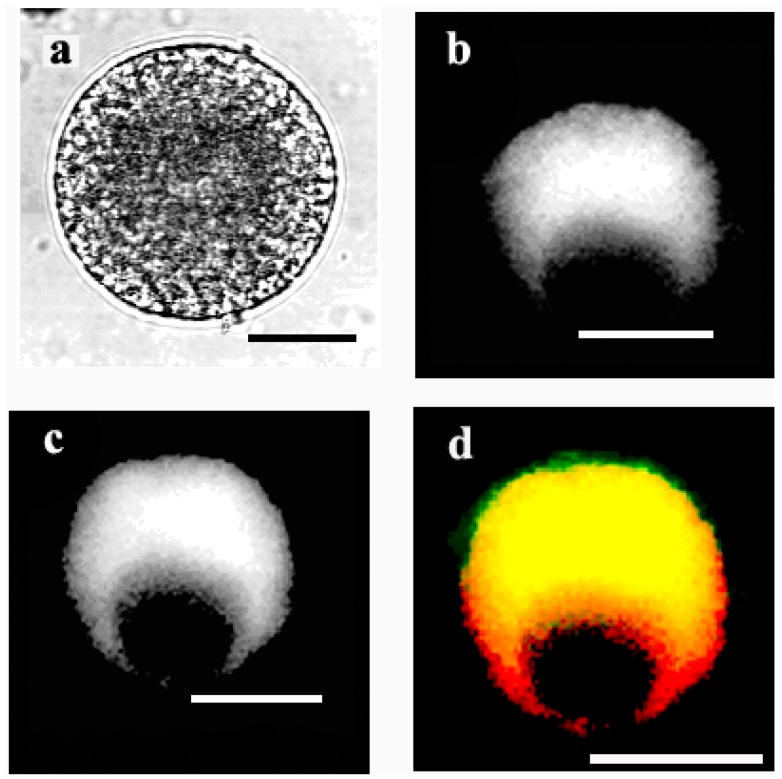
Localization of thapsigargin-sensitive Ca^2+^-ATPase pumps in the wheat egg with fluorescent thapsigargin. (**a**) Transmission-light image of an egg cell; (**b**) stained with fluorescent BODIPY FL^®^ thapsigargin, which was microinjected previously with 1,1'-dihexadecyl-3,3,3',3'-tetramethylindocarbocyanine perchlorate (DiI) to stain the ER membranes visualized in (**c**). (**d**) The overlay image of (**b**) and (**c**). Scale bars: 20, 25, 25 and 25 µm, respectively.

To reveal the localization of thapsigargin-sensitive calcium pumps in the wheat egg cell, the green-fluorescent BODIPY FL^®^ thapsigargin was used. Female gametoplasts were stained with the fluorescence-labelled drug following microinjecting them with DiI (1,1'-dihexadecyl-3,3,3',3'-tetramethylindocarbocyanine perchlorate) (DiIC_16_(3)) which had been previously shown by Pònya* et al.* [36] to selectively label the endoplasmic reticulum membranes in the wheat egg. The images gained of the stained cells support the existence of thapsigargin-sensitive Ca^2+^-ATPase pumps tethered on the membrane meshwork of the endoplasmic reticulum (Figure 8a–d).

**Figure 9:**
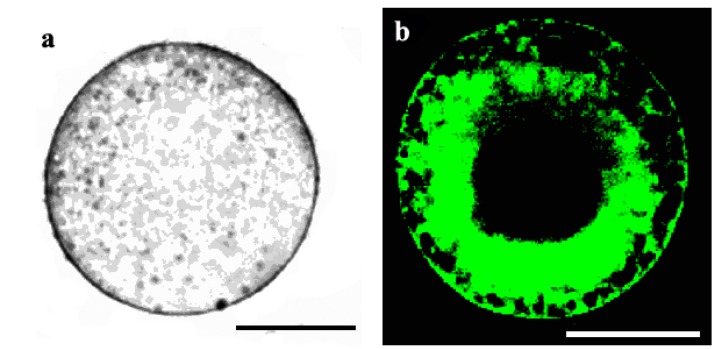
Localization of thapsigargin-sensitive Ca^2+^-ATPase pumps with the green-fluorescent BODIPY FL^®^ thapsigargin applied at a high (100 µM) concentration. (**a**) Transmission-light micrograph of an egg cell stained with BODIPY FL^®^ thapsigargin; and (**b**) imaged using a confocal laser scanning (CLSM) microscope. Scale bars are: 21 and 23 µm, respectively.

When mature egg cells were incubated in the presence of 100 μM BODIPY FL^®^ thapsigargin (applied at the same concentration at which thapsigargin seemed to cause irreversible Ca^2+^ overload sof the treated cells; see Figure 7b), the fluoroprobe markedly stained both the ER and the plasma membrane, suggesting that at this concentration, thapsigargin inhibits the calcium pumps of both the ER and the plasma membrane (Figure 9a,b).

**Figure 10:**
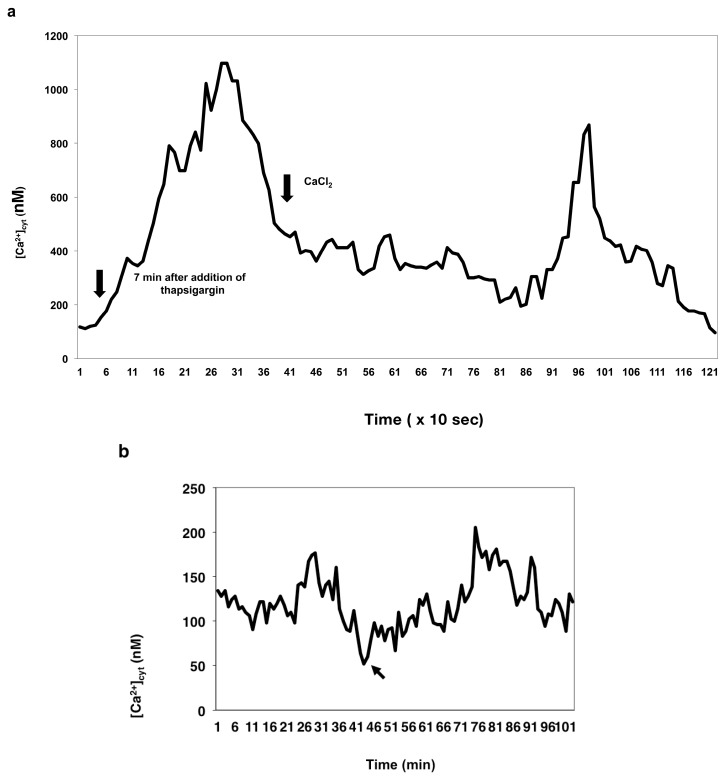
Thapsigargin treatment of isolated wheat egg cells hints at the involvement of an intracellular calcium store in the calcium release mechanism triggered by sperm fusion. (**a**) Thapsigargin activates divalent cation entry in wheat female gametoplasts. The graph represents the change in [Ca^2+^]_cyt_ in an unfertilized wheat egg incubated in thapsigargin in Ca^2+^-free isolation medium followed by the addition of 2 mM CaCl_2_; (**b**) In the control experiment shown, no discernable change was observed in [Ca^2+^]_cyt_ over a 60-min period when 2 mM CaCl_2_ was added to control eggs not previously treated with thapsigargin. The arrow shows the time when thapsigargin was added to the cell.

Thapsigargin activated an influx pathway for Ca^2+^ across the plasma membrane, because a second surge in Ca^2+^ was observed (in 23 out of 28 egg cells; 82.14%) when 2 mM CaCl_2_ was added to eggs previously incubated in thapsigargin in Ca^2+^-free IVF medium (Figure 10a,b).

To examine the effect of thapsigargin on the Ca^2+^ transients at fertilization, mature egg cells treated with 10 μM thapsigargin were fused with sperm cells after [Ca^2+^]_cyt_ had returned to near baseline level (approximately 8 min after adding thapsigargin to the IVF medium).

**Figure 11:**
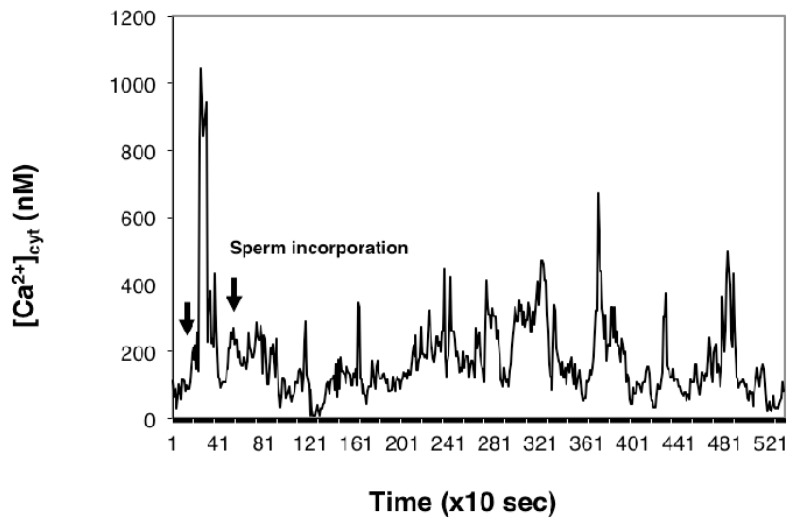
Thapsigargin reduces the amplitude of the sperm-induced transients. Representative [Ca^2+^]_cyt_ measurement in which an egg cell following incubation in IVF medium containing 2 mM CaCl_2_ was treated with 10 μM thapsigargin and fertilized immediately after the thapsigargin-induced transient increase in [Ca^2+^]_cyt_. The first arrow on the left points to the time (7 min) that elapsed from the time of adding thapsigargin to the IVF medium.

As Figure 11 depicts, in egg cells incubated in thapsigargin, the amplitude of the [Ca^2+^]_cyt_ transients (that were observed in mature egg cells fused with the sperm) was substantially reduced.

**Figure 12:**
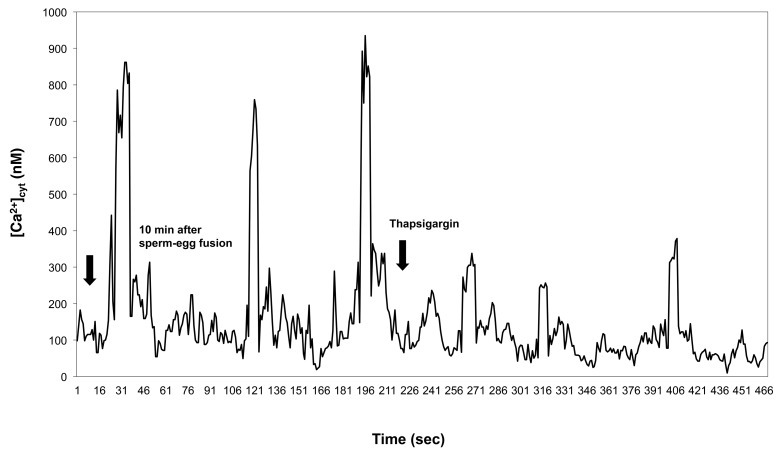
Thapsigargin suppresses [Ca^2+^]_cyt_ transients following sperm–egg fusion. Egg cells were fertilized* in vitro* followed by the addition of thapsigargin after the third sperm-induced [Ca^2+^]_cyt_ transient ensued (the first arrow on the left indicates 10 min post-fertilization).

Thapsigargin added following sperm–egg fusion did not produce an increase in [Ca^2+^]_cyt_ comparable with that triggered by thapsigargin alone; [Ca^2+^]_cyt_ transients continued for some time, and in all cases analyzed (*n* = 25), thapsigargin suppressed the sperm-induced [Ca^2+^]_cyt_ transients observable during fertilization of the receptive wheat egg (Figure 12).

## 3. Discussion

### 3.1. [Ca^2+^]_cyt_ Changes during IVF of Wheat Egg Cells

Antoine* et al.* [41] by simultaneously monitoring extracellular Ca^2+^ flux and [Ca^2+^]_cyt_ by employing the Ca^2+^-vibrating probe and the calcium-sensitive dye fluo-3, found that inhibition of the rise in [Ca^2+^]_cyt_ observed in the maize egg after sperm incorporation prevents both egg activation and a global Ca^2+^ influx, whereas inhibition of the Ca^2+^ influx does not impede [Ca^2+^]_cyt_ elevation nor egg activation. These findings are in agreement with our observation that a single [Ca^2+^]_cyt_ rise appeared sufficient to trigger egg activation in mature wheat egg cells fused with the sperm in calcium-free IVF medium, as demonstrated by cell wall formation. Antoine* et al.* [41] found that the measured Ca^2+^ influx preceded the detected rise in [Ca^2+^]_cyt_. However, when the Ca^2+^ channels in the egg’s plasma membrane were inhibited by Gd^3+^, the sperm cell still could fuse with the egg cell in spite of the blocked Ca^2+^ influx, and the cytoplasmic calcium increase still occurred, presumably due to calcium release from intracellular stores [31]. This observation is important from the angle that it allows the angiosperms to be included in the general model of multicellular organisms in which increased cytoplasmic calcium was shown to be sufficient to induce egg activation [31]. Together with the cytoplasmic calcium increase, the signs of egg cell activation (egg contraction and cell wall deposition) were also observed in Gd^3+^-treated cells [41], indicating that calcium influx is not a prerequisite for creating an increased [Ca^2+^]_cyt_. These authors proposed that the observed calcium influx instead might be needed for sperm incorporation and subsequent karyogamy. This hypothesis implies that *in planta*, the egg cell of flowering plants may have a significant extracellular calcium store at its disposal during fertilization. Indeed, Zhao* et al.* [44] using pyroantimonate precipitation showed that a putative extracellular source of calcium for fertilization could facilitate calcium uptake in the fertilized egg cell of rice via a pool of loosely bound calcium localized in the apoplast of the embryo sac.

Contrary to the observation made by Digonnet* et al.* [26], who measured only a single, long-lasting elevation in [Ca^2+^]_cyt_, our results show that when isolated at the time window of full receptivity, the wheat egg reveals oscillatory changes in [Ca^2+^]_cyt_ upon sperm cell incorporation. Digonnet* et al.* [26] found that there were variations in the duration of the measured Ca^2+^ rise, which they surmised to reflect varying degrees of egg cell maturity, as had been reported by Mòl* et al.* [45]. Thus, the different pattern of calcium dynamics in the fertilized egg cell of maize and that of wheat egg may (at least in part) be explained by the differing maturational stages of the batch of the egg cells and/or by the different methods (calcium-induced fusion* versus* electrofusion) used to induce fusion between the gamete pairs. The possibility that the dynamics of calcium signaling of fertilization has species-specific characteristics in angiosperms cannot be excluded. 

How the observed pulsatile [Ca^2+^]_cyt_ is generated in the fertilized wheat egg cell remains to be elucidated. In the thoroughly studied animal systems, several models were proposed to expound the generation of repetitive [Ca^2+^]_cyt_ rises in non-excitable cells [46,47]. These models differ as to the type of feedback mechanism surmised to regulate Ca^2+^ release and InsP_3_ production and as to whether both Ca^2+^ and InsP_3_ or only Ca^2+^ is thought to oscillate. Whether either of these models can be adopted in explaining the observed [Ca^2+^]_cyt_ changes elicited by sperm incorporation into the wheat female gamete, or different mechanisms are involved, remains to be investigated. In the wheat egg cell, unlike many animal cells and maize, no [Ca^2+^]_cyt_ wave arising at the site of sperm entry was apparent, neither could any local increase in [Ca^2+^]_cyt_ be detected at the sperm entry site (Figure 2a,b). This finding is in agreement with that of Roberts* et al.* [28], who found no evidence for any localized increase in [Ca^2+^]_cyt_ in the *Fucus* egg at the sperm entry site, and suggests a secondary messenger molecule that is responsible for transducing the cue delivered by the sperm cell deep into the cytoplasm-rich region of the egg.

The truncated, spatially-confined [Ca^2+^]_cyt_ waves seen in immature egg cells (Figure 2a) might be explained by an insufficient calcium storage capacity of the intracellular calcium store(s) in immature egg cells. This hypothesis lends credit to the observation of Pònya* et al.* [36] that the Ca^2+^ storage capacity of the ER in the immature egg cell is much less than that in the receptive egg. The “atypical” [Ca^2+^]_cyt_ dynamics revealed by overmature wheat (*T. aestivum*, L.) egg cells (Figure 3c) may be accounted for by a non-functioning (or insufficient) intracellular calcium store, but the possibility of InsP_3_-sensitive channel inactivation caused by protein kinases interfering with coincidence signaling cannot be ruled out (single-cell measurements of protein kinases in egg cells of flowering plants have not been achieved as of yet).

### 3.2. The Possible Origin of the [Ca^2+^]_cyt_ Transients

In the present study, the advantage of electrofusion over calcium-induced fusion [48] (*i.e*., it does not require calcium in the fusion medium to bring about fusion) was exploited in order to establish the origin of the [Ca^2+^]_cyt_ rise and the contribution of extracellular calcium to the observed [Ca^2+^]_cyt_ elevations. This fusion method combined with a pharmacological approach allowed for addressing these questions. First, unfertilized wheat egg cells incubated in calcium-free IVF medium were treated with thapsigargin. Thapsigargin was mainly used in animal systems and shown to block ER/SR Ca-ATPases without interfering with the plasma membrane Ca^2+^-ATPases [37,38]. Its action is based on preventing reuptake from leaky stores, hence elevating [Ca^2+^]_cyt_ in a variety of cell types without stimulating the production of inositol polyphosphates [49,50]. The specificity of thapsigargin action in animal cells is thought to depend on the presence of a recognition site for the inhibitor on all the SR/ER ATPases that is absent from the PM Ca^2+^-ATPase and other P-type ATPases [51]. In contrast, thapsigargin was found to have an inhibitory effect on both ER and PM calcium pumps in isolated membrane vesicles of red beet cells [43]. Our observations, nonetheless, suggest that thapsigargin at a certain concentration range selectively inhibits the transport activity of the ER calcium pump in the wheat egg cell, whereas it has little effect on the plasma membrane Ca^2+^-ATPase. This assumption is based on our findings that:

(1) In the unfertilized egg incubated in IVF medium without extracellular Ca^2+^, thapsigargin at a 10 µM concentration caused a transient increase in [Ca^2+^]_cyt_ which *per se* had to originate from an intracellular calcium store.
(2) Since thapsigargin is an irreversible inhibitor of calcium pumps, the single and rapidly decreasing [Ca^2+^]_cyt_ rise (see Figure 6c,d) suggests that thapsigargin, added at concentrations of 10 and 50 µM, did not interact with the plasma membrane calcium pumps, or if yes, not to the extent that would have prevented them from functioning properly,* i.e.*, pumping the “extra” calcium out of the cytosol; otherwise, the [Ca^2+^]_cyt_ would have remained at a high level for a much longer time (due to the irreversible depletion of both the ER and PM Ca^2+^-ATPase). It may be reasoned, however, that other intracellular Ca^2+^ pumps, such as those located, e.g., in the Golgi apparatus membrane or on vacuole membranes, may have remained unaffected by thapsigargin, which could still facilitate sequestering Ca^2+^ into the Golgi apparatus or into vacuoles. Indeed, Ordenes* et al.* [52] identified thapsigargin-sensitive Ca^2+^ pump activity present in the Golgi apparatus vesicles isolated from the elongation zone of etiolated pea epicotyl.
(3) Nevertheless, imaging of fluorescent thapsigargin-stained egg cells at which the fluorophore was added at a concentration of 10 µM failed to reveal any thapsigargin-binding sites other than those localized in the ER membranes visualized by injecting DiI into the isolated wheat egg cells (see Figure 8a–d). Since imaging Dil injected into wheat egg cells proved to be a reliable and effective method in visualizing specifically the ER membranes in the wheat female gamete [36], this observation argues in favor of our hypothesis. Additionally, Pònya* et al.* [36] identified the ER by CTC (chlortetracycline) labelling to be the main calcium store in the wheat egg cell. Thus, it seems unlikely that calcium leaking from the ER into the cytoplasm upon the addition of thapsigargin could be sequestered into other cell organelles.
(4) The observation that 10 µM thapsigargin treatment caused Ca^2+^ release in unfertilized egg cells incubated in IVF medium without or with calcium and that in the latter case, the [Ca^2+^]_cyt_ transient was not significantly higher compared to that measured when cells were incubated in calcium-free medium (see Figure 6c and Figure 7a) suggests that thapsigargin at this concentration has little effect on the plasma membrane ATPase; otherwise the [Ca^2+^]_cyt_ rise would have been much higher when extracellular calcium was present in the incubation medium due to the cell’s “succumbing” to the tremendous (20,000-fold: 0.1 µM intracellular* versus* 2 mM extracellular Ca^2+^ concentration) “Ca^2+^ pressure” on the cell membrane. In concert with this assumption, when applied to unfertilized egg cells incubated in IVF medium containing 2 mM CaCl_2_, thapsigargin at a high concentration (100 µM) caused a rapidly rising increase in [Ca^2+^]_cyt_, the peak value of which was higher than that observed in wheat egg cells treated with thapsigargin at 10 µM (compare Figure 7a,b). The plateau reached in [Ca^2+^]_cyt_ was sustained during [Ca^2+^]_cyt_ measurement (*n* = 17) and only slightly diminished due to photobleaching of the calcium-sensitive dye. This finding lends credit to the hypothesis that at this concentration, Ca^2+^ overload occurs in the cell, most probably due to the inhibitory effect exerted by thapsigargin on the plasma membrane calcium pumps.

It may, therefore, be concluded that the sensitivity of the ER and the PM calcium pumps to different concentrations of thapsigargin differs in the wheat egg, which may be explained (at least in part) by the proposed mode of action of the compound: thapsigargin, being highly hydrophobic, is believed to partition selectively into the phospholipid component of membranes, where it interacts with the hydrophobic domains of membrane proteins, hence affecting lipid-protein interactions and, consequently, general ATPase activity. The differing degree of sensitivity of the ER and the PM Ca^2+^-ATPase to thapsigargin could, therefore, be attributed to differences in hydrophobicity of the two types of membranes. This notion is corroborated by our finding that fluorescent thapsigargin at a 100 µM concentration stained both the ER and the plasma membrane (see Figure 9a,b).

In the present study, thapsigargin was used to explore the possibility that the first sperm-induced [Ca^2+^]_cyt_ transient acts as a signal for an increase in plasma membrane Ca^2+^ permeability. We examined the hypothesis that the [Ca^2+^]_cyt_ oscillation observed in mature egg cells following fertilization is due to an increased Ca^2+^ permeability and subsequent filling and periodic emptying of an intracellular Ca^2+^ store. The depletion of intracellular Ca^2+^ by thapsigargin in the analyzed egg cells increased Ca^2+^ influx through the cell’s plasma membrane, since an immediate elevation in [Ca^2+^]_cyt_ could be observed when Ca^2+^ was added to unfertilized eggs previously treated with thapsigargin and incubated in IVF medium without extracellular calcium (Figure 10a). Refilling the ER with calcium seems to be a prerequisite for [Ca^2+^]_cyt_ oscillation, as was shown by the suppression of the [Ca^2+^]_cyt_ transients by thapsigargin added to the fusion medium preceding fertilization or after the sperm cell-induced [Ca^2+^]_cyt_ transients ensued (Figure 11 and Figure 12).

These results hint that the repetitive Ca^2+^ transients observed in the mature, fertilized wheat egg protoplasts are produced by the release of Ca^2+^ from the ER that is filled by a thapsigargin-sensitive Ca^2+^ pump. The first sperm-induced [Ca^2+^]_cyt_ transient depletes the ER in the receptive wheat egg and thereby enhances plasma membrane permeability to Ca^2+^. The transient emptying of the ER might then be due to Ca^2+^-induced Ca^2+^ release mediated by an increase in [Ca^2+^]_cyt_ or by the accumulation of Ca^2+^ within the cisternae of the endoplasmic reticulum.

The present study demonstrates that repetitive Ca^2+^ transients in the* in vitro* fertilized, mature wheat egg cell are associated with Ca^2+^ influx across the plasma membrane, which can be suppressed by inhibiting the ability of the endoplasmic reticulum Ca^2+^-ATPase to sequester Ca^2+^. The oscillation in [Ca^2+^]_cyt_ seems to require extracellular calcium, since omitting extracellular calcium from the IVF medium suppressed it. Based on our findings, it might be speculated that *in planta*, the calcium depletion occurring dramatically following sperm–egg fusion in wheat synergids, as was demonstrated by Chaubal and Reger [53], supplies extracellular calcium needed for these repetitive calcium changes seen in the cytoplasm of the fertilized wheat egg cell. It may be hypothesized that calcium signaling, surmised to be involved in the cascade events of signal transduction leading to egg activation, is an event in the fertilized egg cell that sets in within seconds following sperm–egg fusion, hence somehow contributing to the avoidance of polyspermy. However, Digonnet* et al.* [26] observed that adhesion between the two gametes of opposite sexes in the course of fusion brought about by extracellular calcium lasted for 30 min without further membrane fusion, during which time, no variation in fluorescence emission signal occurred. Therefore, it appears that the [Ca^2+^]_cyt_ changes detected in both species (maize and wheat) ensue after a relatively longer “lag period” (30 and 10–12 min in maize and wheat, respectively), rendering it doubtful that [Ca^2+^]_cyt_ in these angiosperm species have (direct) relevance in mechanisms ensuring the avoidance of polyspermy. However, it appears to be clear that in a number of described systems, the intracellular calcium waves induce cortical vesicle fusion with the plasma membrane, leading to the elevation of the so-called “fertilization envelope”, which acts as a mechanical barrier to further sperm penetration, hence indirectly implying changes in [Ca^2+^]_cyt_. This is thought to be a slow block to polyspermy. Whether elevations observed in [Ca^2+^]_cyt_ in the wheat egg have relevance in mechanisms ensuring the avoidance of polyspermy remains to be elucidated. In any case, the difference observed in the two species between the time lapse measured from plasmogamy to the onset of the elevation in [Ca^2+^]_cyt_ may be explained by the different* in vitro* fusion systems used (although surmising a species-dependent variation of the dynamics of intracellular calcium changes cannot be disregarded).

## 4. Experimental Section

### 4.1. Plant Materials

The spring wheat (*Triticum aestivum*, L.) genotype “Siete Cerros” was grown in a growth chamber using a 16-h light period (light intensity: 350 Em^−2^·s^−1^) at 17/15 °C day/night temperature under 70% relative humidity.

### 4.2. Gamete Isolation

Emasculation of the spikes was always carried out precisely when approximately 80% of the microspores were in the late-uninucleate stage. The spikes were harvested at 3, 6, 11, 15 and 18 days after emasculation (DAE), and the ovaries were carefully removed using forceps. Subsequently, the egg cells were isolated from the ovules according to Pònya* et al.* [32]. The isolated cells were individually transferred with a microchip-controlled micropump (A203XVZ, World Precision Instruments, Sarasota, FL, USA) into droplets of mannitol (600 mOsm·kg^−1^), each dispensed in sterile plastic dishes covered with inert oil (voltalef PCTFE oil type 10S, Atochem, Newbury, Berkshire, UK) to avoid evaporation. Following the taking of samples from fresh pollen populations to check their viability [54], sperm cells were isolated using hypoosmotic shock [33] and placed via a micropump system to the egg cells incubated in fusion droplets.

### 4.3. Microinjection of Live Egg Cells and Visualizing the Fluorophores

The microinjection procedure employed to introduce the calcium-sensitive dye, fura-2 dextran (*M*_r_ = 10,000, Molecular Probes, Eugene, OR, USA) into the female gametes has been described in detail by Pónya* et al.* [32]. The injected aliquots of the probe were about 1%–3% of the cell volume estimated by meniscus displacement, assuming the volume of a cone for the tip of the pipette. During the control experiments of the injection procedure, “blind” injections were performed, which were possible by positioning the microneedle in close proximity of the cell surface before switching to the epifluorescence mode of the microscope to launch the [Ca^2+^]_cyt_ measurements. In this manner, the incremental positioning of the micromanipulator arm by the step motor could reproducibly drive the microcapillary into the cytoplasm of the firmly immobilized egg cell. The signal for injection was triggered by pushing a pedal, which generated a “start”-signal recorded by a computer.

### 4.4. The IVF Procedure

The electrofusion of selected pairs of isolated gametes was implemented following the method of Kranz* et al.* [33]. Electrofusion was carried out using a pair of platinum wire electrodes (diameter: 50 μm) on an electrode support controlled by hydraulic microdrives (MO-104, Narishige International Ltd., London, UK). Fusion between the gamete pairs was induced by single or multiple negative DC pulses (50 μs, 0.8 kV·cm^−1^) delivered by a cell fusion instrument (CF-150; BLS—Biological Laboratory Equipment, Budapest, Hungary) following the dielectric alignment of the gamete pairs on one of the electrodes by using an AC field (1 MHz, 75 V·cm^−1^). The fusion droplets were composed of 600 mOsmol mannitol containing 2 mM CaCl_2_ (pH 6.0). Thapsigargin and calcium-containing solutions were directly added by a microsyringe controlled by a micromanipulator to the fusion droplet in an equal volume of medium to ensure rapid and thorough mixing.

### 4.5. Cell Wall Detection

For cell wall visualization egg cells fused with the sperm were stained for 5 min in the dark in the IVF medium containing 0.001% Triton X-100 and calcofluor white M2R (Sigma–Aldrich, St. Luois, MO, USA), then washed twice in a 600 mOsmol/kg mannitol solution before observing them on an inverted microscope (Nikon Eclipse TE-300, Nikon Instruments Europe B.V., Amstelveen, The Netherlands) equipped with epifluorescence.

### 4.6. Measurement of Fura-2 Dextran Fluorescence

Fura-2 dextran-loaded egg cells were observed with a Zeiss Axiovert 35M inverted microscope equipped with a 75-W xenon epifluorescence burner (Osram Licht AG, Munich, Germany). The images were obtained using a Zeiss Plan-Neofluar 63× oil immersion objective (1.25 N.A. (numerical aperture); Carl Zeiss Microscopy, GmbH, Jena, Germany). A rotating filter wheel (Lambda-10, Sutter Instruments, Novato, CA, USA) and a shutter apparatus were used to alternate excitation wavelengths between 340 and 380 nm. A 400-nm dichroic mirror was positioned after the shutter assembly. The emitted light was collected using a 510-nm emission filter with a 10-nm half-bandwidth. Confocal images were produced by using a laser scanning confocal system (Model M-1024, Bio-Rad Microscience Division, Hemel Hempstead, Hertfordshire, UK) coupled with a BX50F4 research microscope (Olympus, Tokyo, Japan). The cells were excited at 488 nm, and the emitted fluorescence was detected at 500–530 nm.

### 4.7. Image Recording and Processing

The images were recorded and digitized using an on-chip integration CCD camera (CoolView, Photonic Science, East Sussex, UK). Switching between the excitation filters was under the control of a computer connected to both the filter wheel and to the camera. The rotating filter wheel was moved to the blank position between each active image capture cycle to minimize photobleaching. Images were captured at a resolution of 256 × 256 pixels and digitized to 256 grey levels. The dark current of the CCD detector was measured prior to each series of images and subtracted during live mode. Autofluorescence was detected at excitation wavelengths of 340 and 380 nm, at which fura-2 has a spectral shift depending on the Ca^2+^ binding/free-acid forms, respectively. Image processing and ratio calculation were performed with the Lucida 3.53 image processing software system (Kinetic Imaging, Ltd., Bromborough, UK). The resulting ratio images were color-coded to represent different calcium concentrations determined after calibration. An* in vitro* (extracellular) calibration of the ratio* versus* free [Ca^2+^] was performed using Ca^2+^-ethyleneglycol-bis(aminoethyl ether)-*N*,*N*'-tetraacetic acid (EGTA) buffers. For the* in vitro* calibration, the buffer contained 0.5 µM fura-2 dextran (*M*_r_ = 10,000), 120 mM KCl, 0.5 mM MgCl_2_, 20 mM 4-(2-hydroxyethyl)-1-piperazineethanesulfonic acid (HEPES) (pH 7.2) and 0.2 mM EGTA (with or without CaCl_2_). To account for the spectral changes of fura-2 fluorescence in cells and in salt solutions, due to viscosity differences [55], 2 M sucrose was added to the calibration buffers in order to increase the viscosity of the standard solutions.

The fluorescent ratio-values were converted into Ca^2+^ concentrations (nM) by using the Grykiewicz, Poenie and Tsien formula [39], [Ca^2+^] = *K*_d_ × [((*R* − *R*_min_)/(*R*_max_ − *R*))*S*_f2_/*S*_b2_], where: [Ca^2+^] = the concentration of calcium ions (nM); *K*_d_ = the dissociation constant of fura-2; *R* = the ratio recorded under appropriate physiological conditions; *R*_min_ = the ratio recorded at zero external calcium; *R*_max_ = the ratio recorded in the presence of excess calcium; *S*_f2_ = the signal at 380 nm in zero calcium; *S*_b2_ = the signal at 380 nm in excess calcium.

### 4.8. Procedure of 1,1'-Dihexadecyl-3,3,3',3'-tetramethylindocarbocyanine perchlorate (DiI) Injection

The microinjection procedure used for introducing DiIC_16_(3) obtained from Molecular Probes (Eugene, OR, USA) into the egg cells was described in detail by Pònya* et al.* [36].

### 4.9. Thapsigargin Treatment

Thapsigargin was purchased from Sigma–Aldrich Chemical Co. (St. Louis, MO, USA) and prepared in a 5 mM stock solution in DMSO, which was then diluted to the appropriate concentrations (0.1–100 µM) in the medium used for IVF.

### 4.10. Visualization of Thapsigargin-Binding Sites in the Wheat Egg

BODIPY FL^®^ thapsigargin was obtained from Molecular Probes (Eugene, OR, USA). For localizing thapsigargin-sensitive Ca^2+^ pumps, egg protoplasts were incubated for 5 min with BODIPY FL^®^ thapsigargin diluted from a stock (1 mg/mL) solution dissolved in DMSO to reach the concentration of 1 µM in the fusion medium. The cells were washed twice before images were acquired through a 40× lens on a Nikon PCM 2000 microscope.

### 4.11. Ca^2+^ Influx Inhibition with Gadolinium

Four microliters of a 1 mM stock aqueous solution of GdCl_3_ purchased from Sigma–Aldrich were added to the fusion medium containing 2 mM CaCl_2_ to obtain a final concentration of 10 µM [30].

### 4.12. Culture Procedures

Following IVF, the fertilized egg cells were transferred to a drop of a modified Kao 90 medium consisting of Kao 90 solution [56] supplemented with zeatin (1 mg·L^−1^) adjusted with mannitol to 600 mOsmkg^−1^ and solidified with 1% (*w*/*v*) low-melting point agarose. To enhance the elongated growth of the fusion products (see [40]), 5 µM naphthalene 1-acetic acid (NAA, auxin, Sigma–Aldrich Chemical Co., St. Louis, MO, USA) was added to the medium on the third day of* in vitro* culture. The imaged fusion products were placed in 12-mm transwell inserts (Costar Corporation, Cambridge, MA, USA), hanging on the rims of 12-well dishes containing 1 mL of a microspore suspension from the winter barley cultivar “Igri”. The cultured cells were immobilized on a thin alginate layer.

## 5. Conclusions

Taken together, our experimental results suggest that the strategy of the wheat female gamete for altering its cytoplasmic calcium level relies on an intrinsically more stable mechanism of calcium homeostasis (calcium storage performed by the ER, instead of relying exclusively on calcium influx through the plasma membrane). Furthermore, the repetitive [Ca^2+^]_cyt_ elevations in the mature angiosperm female gamete activated by the sperm cell appears to be comparable to the dynamics of [Ca^2+^]_cyt_ oscillations triggered by an oscillation-inducing sperm protein (“oscillogen”) demonstrated to induce a characteristic series of Ca^2+^ oscillations in the mammalian egg at fertilization [57]. It seems that certain Ca^2+^ signatures identified in widely differing systems transcend the specific system that they are found in [58]. For instance, some fertilization-related Ca^2+^ wave signatures appear conspicuously similar [58]. Calcium fluctuations were also reported in tobacco central cells [59]. The findings presented here supply evidence that the spatial and temporal changes in [Ca^2+^]_cyt_ may represent the initial steps in egg cell activation during fertilization in higher plants (for a review, see [60]).

Our observations may have ramifications for enhancing our understanding of the mechanisms of double fertilization in angiosperms with particular regard to the presumable interaction between the synergids and the egg cell during pollen tube penetration and discharge [61].

## Supplementary Materials

Supplementary materials can be found at http://www.mdpi.com/1422-0067/15/12/23766/s1.
